# Molecular mechanisms of macrophage Toll-like receptor–Fc receptor synergy

**DOI:** 10.12688/f1000research.12679.1

**Published:** 2018-01-08

**Authors:** Michelle Lennartz, James Drake

**Affiliations:** 1 Department of Regenerative and Cancer Cell Biology, Albany Medical College, 47 New Scotland Avenue, Albany, NY, 12008, USA; 2 Department of Immunology and Microbial Disease, Albany Medical College, 47 New Scotland Avenue, Albany, NY, 12008, USA

**Keywords:** Macrophages, toll-like receptors, Fc receptors

## Abstract

Macrophages (MØs) are a key cell type of both the innate and the adaptive immune response and can tailor their response to prevailing conditions. To sense the host’s status, MØs employ two classes of receptors: Toll-like receptors (TLRs), which are sensors for pathogen-derived material, and Fcγ receptors (FcγRs) that are detectors of the adaptive immune response. How MØs integrate the input from these various sensors is not understood and is the focus of active study. Here, we review the recent literature on the molecular mechanisms of TLR and FcgR crosstalk and synergy, and discuss the implications of these findings. This overview suggests a multilayered mechanism of receptor synergy that allows the MØ to fine-tune its response to prevailing conditions and provides ideas for future investigation.

## Introduction

Macrophages (MØs) are among the key effector cells of host defense and have the ability to tailor their response to the host’s immune status. To accomplish this feat, MØs use two classes of immune receptors: Toll-like receptors (TLRs), which are sensors that drive the innate immune response, and Fcγ receptors (FcγRs) that are detectors of the adaptive immune response. How MØs integrate the inputs from these two distinct signaling pathways is not fully understood and is the focus of active investigation.

Much of our knowledge of the signaling pathways activated by TLR and FcγR has been determined from *in vitro* studies directly engaging single receptor types with saturating doses of strong ligands (for example, lipopolysaccharide [LPS] and immune complexes). However, during infection, it is likely that conditions will lead to less than 100% receptor engagement, meaning that physiologically MØs will be responding to submaximal receptor signaling. Moreover, the presence of natural antibodies able to opsonize incoming pathogens ^1– 3^ suggests that most pathogens will engage both TLR *and* FcγR, even in naïve hosts. As the host response is thus driven by the integration of the TLR and FcγR inputs, our comprehension of the mechanisms of TLR–FcγR crosstalk is critical to our understanding of host defense.

In broad terms, in the issue of receptor signal integration, crosstalk between the two receptor-driven signaling pathways could regulate messenger RNA (mRNA) transcription or mRNA translation into protein or both. In the case of transcriptional control, integration of TLR and FcγR signals would regulate the levels and activity of transcription factors that control the production of cytokine mRNA. Alternatively, signal integration could occur post-transcriptionally, whereby signals from either of the two pathways would modulate the efficiency of mRNA translation. Because of the need for tight regulation of MØ activation and cytokine production, it would not be surprising if both pre- and post-transcriptional mechanisms were at work.

Although the mechanisms of TLR–FcγR signal integration are incompletely understood, recent advances provide clues as to how MØs might integrate these signals for the tight regulation of cytokine production. With respect to FcγR, we will focus on crosstalk involving the activating (as opposed to inhibitory) FcγR.

## Pre-transcriptional control

Although it is well accepted that there is crosstalk between the TLR and FcγR signaling pathways, the underlying cellular and molecular mechanisms are far from clear. A primary signaling pathway for most TLR is through MyD88, TIRAP, and TRAF6, which ultimately leads to the activation of transcription factors such as nuclear factor kappa B (NF-κB) and activator protein 1 (AP-1) ^4^. In contrast, signaling by activating FcγR occurs via src family kinase-mediated phosphorylation of immunoreceptor tyrosine-based activation motifs (ITAMs), which results in the recruitment of the tyrosine kinase Syk for triggering of downstream pathways, including activation of phospholipase C, generation of an intracellular calcium flux, and NADPH activation ^5, 6^.

Even though there are shared signaling molecules between the two pathways, how these two distinct signaling tracks communicate is not completely clear. Interestingly, a recent unbiased analysis of the TLR2/4-induced phosphoproteome in MØs revealed multiple targets within the FcγR-mediated phagocytosis pathway that are phosphorylated in response to TLR2/4 ligation and demonstrated an ability of TLR2/4 signaling to augment FcR-mediated phagocytosis ^7^, suggesting multiple nodes for TLR regulation of FcγR signaling.

Although TLR–FcγR crosstalk may occur at the level of shared downstream signaling molecules such as MAPK and components of the NF-κB pathway, it is also possible that the interaction occurs more proximal to the receptor at the level of Syk activation. Though the role of Syk in FcγR signaling is well known, Syk’s involvement downstream of TLR engagement is less well appreciated. The first evidence linking Syk and TLR was from Arndt and colleagues, who reported the LPS–TLR4-driven activation of Syk in neutrophils ^8^. Here, the authors were able to co-immunoprecipitate TLR4 and Syk ^8^, suggesting either a direct interaction between the two molecules or the presence of a signaling complex. Subsequent publications have demonstrated a similar TLR–Syk interaction in MØs (for example, 9– 12). Interestingly, TLR4 activation of Syk in MØs and other cells seems to be indirect in that it requires the TLR to act, in an undefined way, through an ITAM-containing protein such as Dectin-1/DAP12 or the FcR γ-chain ^11, 12^. In the context of IgG-opsonized pathogens, FcγR, rather than DAP12, would likely provide the ITAM for Syk activation ^13^. As TLR4 and its co-receptor CD14 as well as the activating FcγRs reside in lipid micro-domains colloquially termed “lipid rafts” ^14, 15^, these rafts could promote receptor synergy by bringing the two types of receptor into close physical proximity. TLR residence within lipid rafts is driven by ligand binding and is thought to be mediated by cholesterol or sphingolipid binding motifs (or both) in, or immediately adjacent to, the TLR transmembrane domain (reviewed in 15). Ligand-induced TLR raft recruitment is thought to then favor TLR interaction with downstream signaling molecules such as MyD88 and TIRAP to facilitate TLR signaling ^14, 15^. Moreover, recent studies of TLR4 signaling have revealed that monoclonal antibody-mediated co-ligation of TLR4 and FcγR can result in co-recruitment of the tethered receptors to lipid rafts and alteration of “normal” TLR4 signaling ^16^. Consistent with this report, our work suggests that remodeling of MØ lipid rafts using either methyl-β cyclodextrin (MβCD) or filipin profoundly changes (that is, increases or decreases, respectively) the MØ interleukin-6 (IL-6) cytokine response to TLR2–FcγR co-engagement (D. Hunt and J.R. Drake, unpublished data). Thus, although there is intriguing evidence that TLR–FcγR crosstalk may occur at a receptor proximal stage of signaling, perhaps through assembly of signaling platforms within lipid rafts, the molecular basis for that interaction and the mechanism of TLR–FcγR–Syk communication require much additional investigation.

FcγRs are not the only ITAM-bearing receptors that can interact with TLR, as both the B-cell receptor (BCR) on B lymphocytes (discussed below) and IgE FcR on basophils/mast cells (recently reviewed in 17) have been shown to interact with multiple TLRs. Information gained from studies of TLR–BCR interactions is particularly relevant to the consideration of TLR–FcγR synergy. Foundational work by the Marshak-Rothstein lab revealed a synergy between the BCR and the MyD88-linked TLR7 and TLR9 ^18, 19^. Subsequent studies, recently summarized by Suthers and Sarantopoulos ^20^, have identified several potential points of communication between TLR signaling pathways and BCR ITAM signaling. For example, one aspect of TLR–BCR crosstalk involves Syk-driven upregulation of the TLR signaling molecule TRAF6 ^21^. However, this mechanism requires induction of new gene expression, a process that would take minutes to hours and thus cannot account for receptor synergy that occurs within seconds of receptor engagement. However, a recent publication by Schweighoffer and colleagues ^22^ revealed a faster-acting potential molecular mechanism. Here, it appears that in B cells TLR4 engagement drives Syk activation *through the BCR* (in the absence of a BCR ligand) via a mechanism that does *not* involve MyD88. Thus, ligand binding to the TLR is influencing the neighboring unligated BCR via an unknown mechanism to drive Syk activation. It is possible that a similar mechanism underlies TLR–FcγR synergy.

Regardless of whether lipid rafts are involved in the signaling interactions between TLR and FcγR/BCR, it is also possible that crosstalk involves intermediary proteins that facilitate communication between the TLR and the ITAM-bearing receptor. One candidate for such an intermediary is the protein SCIMP (SLP adaptor and CSK interacting membrane protein). SCIMP is a member of the family of transmembrane adaptor proteins (TRAPs) and is known to be involved in Src family kinase signaling in B cells ^23^. A recent study found that knockdown of SCIMP in MØs results in an inhibition of signaling by TLRs 2, 3, 4, and 7. Interestingly, upon ligand-binding, SCIMP associates with TLRs in a MyD88-independent fashion. Although SCIMP itself is *not* an ITAM-bearing protein, SCIMP-associated Src kinase molecules could mediate phosphorylation of the ITAMs of TLR-associated FcγR/BCR molecules, driving subsequent Syk recruitment and activation. In addition, SCIMP is enriched in lipid rafts ^23^, suggesting that the mechanisms of TLR–FcγR synergy may involve *both* intermediary membrane proteins such as SCIMP *and* membrane lipid micro-domains such as lipid rafts.

Taking all of this into consideration, we would argue that TLR ligation drives two distinct downstream signaling pathways. One is the canonical MyD88-dependent pathway that leads to the activation of NF-κB. The second is a Src–Syk-dependent pathway downstream of an ITAM. In this model, the strength of the MyD88 versus ITAM inputs would tailor the cell’s ultimate response. “Strength of signal” can vary in response to changes in parameters such as the percentage of bound receptors (along an infection continuum where the number of pathogens follows a bell curve), the degree of pathogen opsonization, the avidity/affinity of the TLR for the pathogen-associated molecular pattern (PAMP) or FcγR for the opsonizing IgG subtype or the size of the particle or both. For example, conditions of partial TLR engagement, such as would occur at the early stages of infection with few bacteria avidly binding to TLR but only low circulating IgG levels, could lead to robust activation of the MyD88-dependent arm of TLR signaling but weak activation of Syk-dependent signaling. This would lead to partial/incomplete MØ activation. In contrast, MØ interaction with the same pathogen opsonized with many IgG molecules (at the peak of infection when IgG levels are high) would promote TLR–FcγR synergy to drive robust MØ activation. Here, bacterial PAMPs such as LPS would engage the TLR to drive MyD88-dependent signaling, while IgG binding to FcγR would directly drive ITAM-dependent Syk signaling. This model of TLR–FcγR synergy in MØs is derived from information gained from many experiments using disparate cell types and receptor agonists and provides a framework for future studies.

## Post-transcriptional control

Regulation of mRNA translation/stability is a general mechanism to fine-tune cellular responses such as cytokine production, and microRNAs (miRNAs) are one tool that the cell can use to this end. Although to the best of our knowledge there are no published studies on the role of miRNA in the crosstalk between MØ TLR and FcγR, recent reports on the interplay between miRNA and TLR/FcγR signaling raise some interesting possibilities for future investigation. Here, we will discuss those findings in a general framework of MØ IL-6 and tumor necrosis factor-alpha (TNF-α) production, as these cytokines are two key MØ-produced inflammatory mediators.

miRNAs are short noncoding RNAs of about 21 nucleotides in length and can regulate protein production in multiple ways. In addition to binding mRNA molecules and targeting them for degradation via the RNA-induced silencing complex (RISC), miRNAs can interfere with mRNA translation as well as nascent polypeptide stability ^24, 25^.

When MØs are activated with strong TLR ligands such as purified *Escherichia coli* LPS, numerous miRNAs are downregulated ^26^, including those that inhibit production of the inflammatory cytokines TNF-α (miR-24) and IL-6 (miR-24 and Let-7c) ^27, 28^. Consequently, robust TLR signaling would lead to *both* the upregulation of cytokine mRNAs via a MyD88/NF-κB-dependent pathway *and* the downregulation of miRNAs that would block the translation of cytokine mRNA into protein. One of the questions raised by this observation is how these miRNAs might block cytokine protein production. Here, recent work from the Nares lab suggests an interesting possibility ^29^. Their report shows that, in human monocytic cells, ectopic overexpression of a miR-24 mimetic results in a significant decrease in both TNF-α and IL-6 cytokine production but *no* downregulation of either mRNA species. This indicates that the blockade in inflammatory cytokine production is most likely occurring via a miRNA-based inhibition of mRNA translation. In regard to the role of miRNA in TLR signaling, it is currently unclear whether the signals that elicit TLR-driven miRNA downregulation are MyD88 or Syk dependent. However, the finding that FcγR signaling can also lead to the downregulation of the same miRNA species (see below) suggests that it may be the Syk-dependent pathway.

Under physiological conditions of TLR engagement, such as MØ interaction with an individual bacterium, it is unclear whether the levels of induction of cytokine mRNA and downregulation of miRNA would be similar. It is possible that some conditions of TLR engagement lead to robust cytokine mRNA induction but little or no downregulation of the miRNA that would block the translation of these mRNA molecules, resulting in little or no cytokine production. Under these conditions, TLR–FcγR synergy could become very important to the development of a robust cytokine response. In human monocyte-derived MØs, FcγR signaling has been shown to downregulate the level of miR-24, which is known to block the translation of IL-6 and TNF-α mRNA ^28, 30^. In addition, it has been shown in tumor cells that Src kinase signaling can drive the downregulation of Let-7a (which can also block IL-6 cytokine production) ^31^, suggesting that FcγR ITAM Src signaling in MØs might also downregulate levels of Let-7 miRNAs. This would mean that MØ interaction with even low numbers of antibody-opsonized bacteria could result in robust inflammatory cytokine production. Here, TLR signaling would be responsible for driving the transcription of cytokine mRNA molecules, while FcγR ITAM signaling (driven by FcγR recognition of the opsonizing antibodies) would downregulate miR-24/Let-7 miRNA to allow efficient cytokine mRNA translation. Hence, TLR–FcγR synergy may be occurring at a post-transcriptional stage in the cytokine response and be mediated by miRNA. However, this intriguing possibility requires additional investigation.

## Conclusions

The ability of MØs to integrate and respond to multiple signals from their ever-changing environment is critical for MØ function and the health of the host. Although it is clear that TLR and FcγR are two key MØ receptors that allow the cell to discern the immune state of the host, the molecular mechanisms that underlie this feat are incompletely understood. At the pre-transcriptional step, there are multiple nodes of interaction between the TLR–MyD88 signaling pathway and FcγR–ITAM–Syk signaling pathways that represent potential points of crosstalk. At the post-transcriptional step, TLR and FcγR regulation of inhibitory miRNA are also likely involved. This sophisticated regulatory web would allow precise tailoring of the MØ response to environmental cues. Dendritic cells also express TLR and FcR, and it is known that crosstalk occurs in these cells as well. However, the underlying molecular mechanisms and impact of crosstalk on immune function appear to be different (recently reviewed in 32). Future studies on the precise underlying molecular mechanisms in both cell types will yield exciting new insights.
